# Prognostic Factors and Outcome of Patients with Adult Acute Lymphoblastic Leukemia Treated with the Hyper-CVAD Regimen: A Retrospective Study

**DOI:** 10.1155/2023/5593635

**Published:** 2023-11-09

**Authors:** Zahra Malakoutikhah, Farzaneh Ashrafi, Ali Derakhshandeh

**Affiliations:** ^1^Applied Physiology Research Center, Cardiovascular Research Institute, Isfahan University of Medical Sciences, Isfahan, Iran; ^2^Department of Internal Medicine, School of Medicine, Isfahan University of Medical Sciences, Isfahan, Iran

## Abstract

**Aim:**

The Hyper-CVAD regimen has shown promising results for adult patients with acute lymphoblastic leukemia (ALL), as designed by the MD Anderson Cancer Center (MDACC). This treatment has resulted in a complete remission rate of 92% and a 5-year overall survival of 38%. However, given the diversity of patient demographics and institutional methods, outcomes may differ between various institutions. This study will compare the outcome of adult ALL patients treated with the Hyper-CVAD regimen in Iran with those obtained in the original series presented at the MDACC. *Patients and Method*. In this retrospective study, we evaluated the 2-year leukemia-free survival (LFS) and the 2-year overall survival (OS) of 70 ALL patients treated between 2014 and 2019 in the Seyed Al-Shohada Hospital in Isfahan, Iran.

**Results:**

In total, 59 ALL patients (84.28%) achieved complete remission (CR). The CR rate had statistical differences by bone marrow transplantation (BMT) and WBC count. The 2-year LFS and OS were 40% and 42%, respectively. There were significant differences in LFS and OS by BMT, myeloid marker, and WBC count.

**Conclusion:**

The outcome of the traditional Hyper-CVAD regimen in treating adult ALL was not satisfying. More efficient therapies should be applied for the treatment of adult ALL.

## 1. Introduction

Acute lymphoblastic leukemia (ALL) is an aggressive hematological malignancy in which the normal process of hematopoietic cell maturation is disrupted. The lymphoid progenitor cells are suppressed at an early stage of differentiation, proliferating rapidly and replacing the bone marrow's normal hematopoietic cells. The worldwide annual incidence of ALL is approximately one case in every 100,000 people [1]. The incidence of ALL peaks aggressively in children aged one to four years then peaks again among adults from the age of 50. Although ALL is the most common malignancy in children, it is rare in adults, accounting for one percent of all adult cancers. The treatment of childhood ALL is a real success story in oncology, resulting in a highly curable disease with long-term survival rates (>90%). However, the ALL survival rate decreases sharply with age, and adult acute lymphoblastic leukemia has a poor prognosis with a high mortality rate [2]. Although most patients reach complete remission following the initial chemotherapy treatment, the cure rate of adult ALL is as low as 20–40% and most of them finally develop relapse [3]. Hence, the management of ALL in adult patients is still challenging. Evidence exists to show that more aggressive chemotherapeutic regimens for ALL have improved the survival rate. The dose-intensive regimen known as Hyper-CVAD (hyperfractionated cyclophosphamide, vincristine, doxorubicin, and dexamethasone)/HD MTX-Ara-C (high-dose methotrexate and cytarabine) was established in 1992 and has since become the standard of care for ALL. This treatment plan involves eight cycles of intensive Hyper-CVAD therapy alternated with HD MTX and Ara-C, followed by 2.5 to 3 years of maintenance therapy.

Kantarjian and colleagues from MD Anderson Cancer Center (MDACC) came up with this treatment plan that adopted the fundamental therapeutic principles of pediatric regimens. However, this regimen relied less on asparaginase during remission induction [4]. The outcome of patients treated with the Hyper-CVAD regimen has been promising, with a complete remission (CR) rate of 92% and 5-year overall survival (OS) of 38% [5].

However, this regimen's feasibility and results may vary depending on the center, the frequency of poor prognosis ALL, and exclusion criteria. This study compares the outcome of adult ALL patients treated with the traditional Hyper-CVAD regimen in Iran with those obtained in the original series presented at MDACC.

## 2. Patients and Method

### 2.1. Study Design

A single-center retrospective study was carried out at the Seyed Al-Shohada Hospital in Isfahan, Iran, a tertiary care hospital focusing on hematological malignancies. The ethical committee of Isfahan University of Medical Sciences approved the current study (ethical number: IR.MUI.MED.REC.1400.332).

Seventy consecutive adult patients with ALL treated with the Hyper-CVAD regimen were enrolled in this retrospective study between July 2014 and July 2019. Eligibility criteria included ALL diagnosis, ages above 15, adequate clinical information in the patient's record, and contact information to make a call if needed. Moreover, the coexistence of other malignancies or lack of patient consent was considered exclusion criteria. The diagnosis of ALL was based on bone marrow morphology and immunophenotyping. Demographic, laboratory, and clinical data were collected from institutional databases that were continuously updated. If the patient's data were not wholly registered, the phone number indicated in the file would be contacted. After explaining the purpose of the study and getting permission, they will be asked the desired questions.

### 2.2. Statistical Analysis

Data were analyzed using the SPSS version 21.0 Statistical package (SPSS Inc., Chicago, IL, USA). Qualitative and quantitative data were presented as frequency (percentage) and mean ± SD, respectively. Descriptive statistics were applied to explore and describe the data. Data were analyzed by Chi-square test, *t*-test, Fisher exact test, Mann–Whitney test, and Kaplan–Meier survival analysis.

## 3. Results

### 3.1. Patients

This study included 70 ALL patients treated with the Hyper-CVAD regimen.  Table 1 displays the characteristics of the patients, including a male-to-female ratio of 1.33 (with 40 male and 30 female patients). The average age at diagnosis was 36.61 ± 13.88 years, with a median age of 36.5 [16–73]. Most patients (77.1%) had B-cell ALL, while 22.9% had T-cell ALL. Interestingly, 5.71% of patients showed primary CNS involvement. Karyotype or molecular analysis revealed that 18.6% of patients were Philadelphia positive and 10% had myeloid markers (CD13, CD33). Additionally, 22.8% of patients underwent bone marrow transplantation (BMT) after receiving chemotherapy. The mean ± SD of WBC and LDH was 42 ± 90 × 10^9^/L and 1905 ± 1765, respectively.

### 3.2. Treatment Results

Out of the 70 patients observed, 59 (84.28%) achieved complete remission (CR). The data indicated that BMT (*p* =0.040) and WBC count (*p* =0.033) had statistical differences in CR rate. However, gender, cell lineage, BCR/ABL translocation, myeloid markers, or LDH count did not show significant differences in CR rate. During the remission phase, 25 (42.3%) patients experienced bone marrow (BM) relapse while 10 (16.9%) patients faced CNS relapse. Among the 10 patients with CNS relapse, 5 had isolated CNS relapse, 3 encountered CNS relapse following BM relapse, and 2 had simultaneous BM and CNS relapse.

### 3.3. Survival Analysis

**Figure 1:**
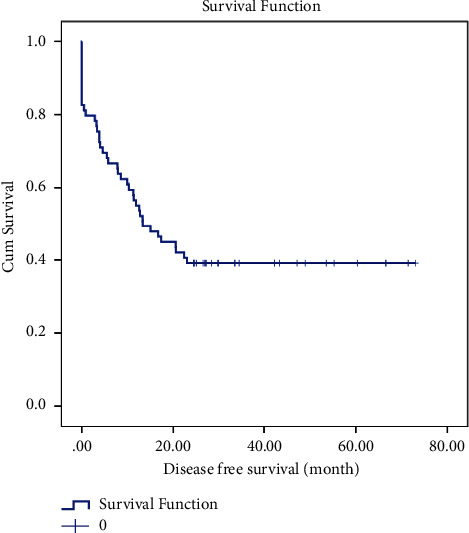
Leukemia-free survival of ALL patients treated with the Hyper-CVAD regimen.

**Figure 2:**
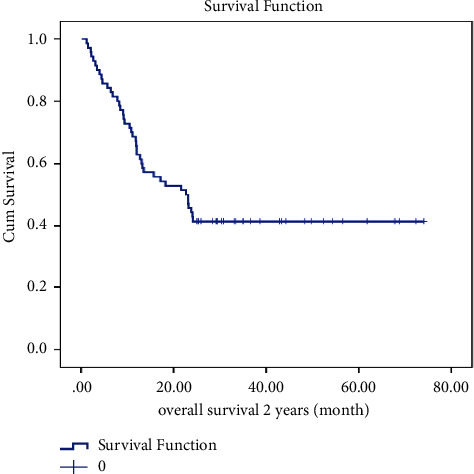
Overall survival of ALL patients treated with the Hyper-CVAD regimen.

The leukemia-free survival (LFS) curve of patients is shown in Figure 1. The mean ± SD LFS time for this study was calculated to be 32.971 ± 3.920 months, and the median ± SD LFS time was found to be 13.300 ± 3.263 months. One-year LFS was 51%, and 2-year LFS was 40%. There were statistical differences in LFS by BMT (*p* =0.030), myeloid marker (*p* =0.041), and WBC count (*p* =0.017). However, there were no significant differences in LFS by gender, cell lineage, presence of BCR/ABL translocation, or LDH count. Moreover, as illustrated in Figure 2, the estimated mean ± SD OS in ALL patients was 37.31 ± 3.74 months and the median ±SD OS was 22.53 ± 4.2 months. One-year OS was 61%, and 2-year OS was 42%. Statistically significant differences were observed in OS based on BMT (*p* =0.026), myeloid marker (*p* =0.042), and WBC count (*p* =0.025). However, there were no notable statistical differences in OS based on gender, cell lineage, presence of BCR/ABL translocation, or LDH count.

**Figure 3:**
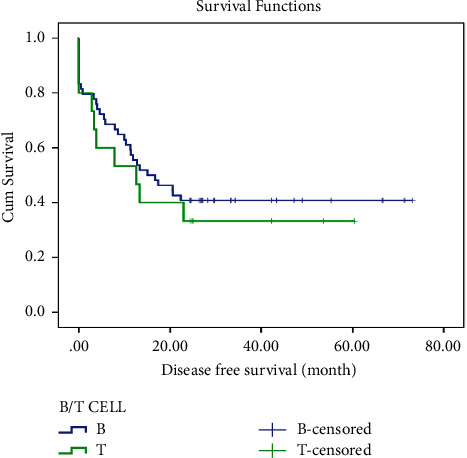
Leukemia-free survival of ALL patients by the type of malignancy.

**Figure 4:**
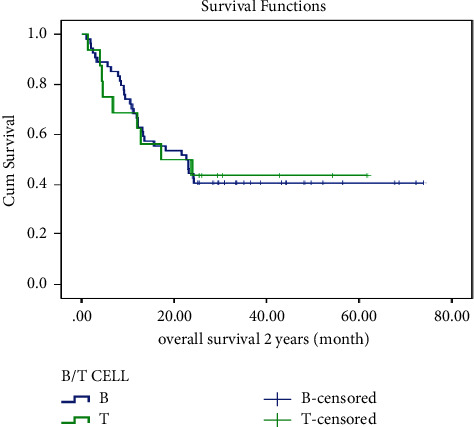
Overall survival of ALL patients by the type of malignancy.

In this study, LFS and OS were also evaluated by cell lineage and BMT. The mean ± SD LFS was estimated to be 34.1 ± 4.4 in B-cell ALL and 24.5 ± 6.7 in T-cell ALL. The 2-year LFS was 40% in both groups. Similarly, the estimated mean ± SD OS in B-cell ALL was 37.1 ± 4.2, compared to 32.4 ± 6.6 in T-cell ALL. The two-year OS in the B-cell group was 41%, while in the T-cell group, it was 46%. Thus, there were no significant differences in LFS and OS by cell lineage, as shown in Figures 3 and 4.

**Figure 5:**
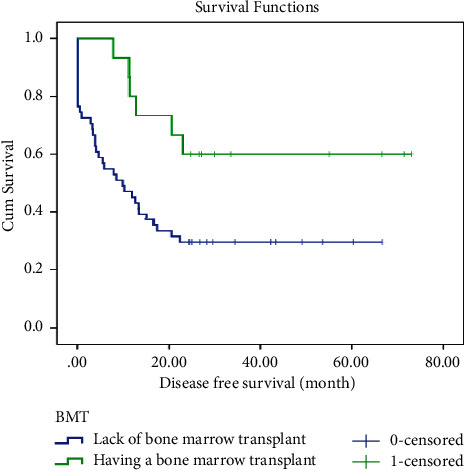
Leukemia-free survival of ALL patients by the BMT.

**Figure 6:**
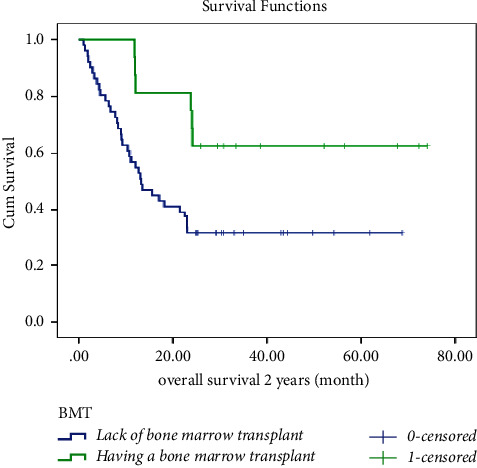
Overall survival of ALL patients by the BMT.

However, evidence shows a considerable difference in LFS and OS based on BMT. BMT resulted in 49.5 ± 7.4 months LFS and 52.9 ± 6.8 months OS, whereas the lack of BMT resulted in 23.8 ± 3.9 months LFS and 28.5 ± 3.8 months OS. The 2-year LFS and OS in the BMT group were both 62%, while in the non-BMT group, they were 29% and 31%, respectively (Figures 5 and 6).

## 4. Discussion

Differences in ALL biology, comorbidities, and patient tolerance make the adult ALL more complicated with poorer outcomes than their pediatric counterparts. Despite novel therapies developed for ALL, the outcome of patients in Iran is still disappointing and has not changed significantly over the last decades. This condition may be due to the poor survival of adult ALL patients, relapses without apparent reason, and high treatment costs even in the remission period [6]. More intensive chemotherapy regimens have been shown to have a better outcome in adult ALL patients. One of the most popular chemotherapeutic regimens for the treatment of adult ALL is presently Hyper-CVAD. The Hyper-CVAD regimen was first developed in the MDACC with CR rates of 92% and a 5-year OS of 38% [5].

We compared the results of the Hyper-CVAD regimen in the Iranian population with the previous studies. The prognostic factors observed in the present study showed a similar trend to those reported in other studies that evaluated Hyper-CVAD efficiency. However, according to novel therapeutics efficiency, the outcome of the Hyper-CVAD regimen is no longer satisfying and overall survival is poor. Our observations indicated that 59 out of the total patients (84.2%) achieved CR, which is consistent with the findings of the Buyukasik (84.2%) [7] and the Mehrzad (84%) [8] studies, higher than that reported in the Jalaeikhoo et al. (81.7%) [6] and Xu et al. (73.6%) [9] studies. However, our study's CR rate was lower than most studies, with CR rates higher than 90% [1, 3–5, 10, 11]. The 2-year LFS and OS in our study were notably lower than those reported in similar studies, at 40% and 42%, respectively. However, these results are consistent with the 5-year LFS and OS reported in the original series presented at the MDACC, as well as other comparable studies [1, 3–5].

Variations in the research demographics and the efficacy of the salvage treatments used may be responsible for this discrepancy. However, induction mortality, infectious complications, and high rates of relapse in some populations generally limit the traditional Hyper-CVAD regimen. The initial Hyper-CVAD regimen has undergone several revisions since its initial development to optimize it for various patient demographics and safely combine innovative therapies without compromising tolerability. Implementing adjustments in maintenance and intensification, along with the inclusion of innovative monoclonal antibodies, BCR::ABL1 tyrosine kinase inhibitors (TKIs), and the utilization of growth factors, has led to substantial improvements in patient outcomes [12].

Monoclonal antibodies such blinatumomab [13], rituximab [14], ofatumumab [15], and inotuzumab [16, 17] have demonstrated enhanced response rates, better survival outcomes, and reduced side effects and toxicities when employed as part of the initial therapy regimen in combination with Hyper-CVAD. The pairing of Hyper-CVAD with blinatumomab (a bispecific CD19 antibody) yielded an 85% CR rate at the two-year mark and halved the duration of maintenance courses, reducing it from 30 months to 15 months [18]. Elderly patients could receive a reduced treatment dosage and need fewer maintenance cycles, decreasing from three to one, yet they still achieved a remarkable 96% overall response rate with the mini-Hyper-CVAD regimen (with lower intensity and excluding anthracycline) combined with inotuzumab (CD19 antibody) and optionally blinatumomab [17]. Hyper-CVAD in combination with ofatumumab was found to be significantly superior to Hyper-CVAD combined with rituximab, resulting in a 3-year overall survival (OS) rate of 98% [15]. Moreover, the use of blinatumomab in combination with dasatinib for newly diagnosed Philadelphia chromosome-positive acute lymphoblastic leukemia (Ph + ALL) resulted in a 60% molecular response (MR), including a 41% complete molecular response (CMR) and an 88% OS rate at 24 months [19].

The integration of BCR::ABL1 tyrosine kinase inhibitors (TKIs) alongside intensive chemotherapy represented a significant milestone in the treatment of Ph + ALL. The development of second and third-generation TKIs has further enhanced the prospects for extended survival in individuals with Ph + B-ALL, illuminating the unique complexities associated with this specific type of leukemia [20]. The inclusion of ponatinib, a third-generation pan-BCL-ABL inhibitor, has resulted in a remarkable 100% CR rate among patients with Ph + ALL [21]. Furthermore, the necessity for allogeneic stem cell transplantation (SCT) is found to be uncommon in these patients. Ponatinib, a remarkably potent tyrosine kinase inhibitor, has demonstrated outstanding effectiveness in managing T315I mutations, as documented by Sasaki et al. in 2016. As a result, the combination of Hyper-CVAD chemotherapy with ponatinib has emerged as the established standard of care for patients diagnosed with Ph + ALL [22].

In our study, the mean ± SD of WBC count was 42 ± 90 × 10^9^/L, which had significant differences in responders (27 ± 57 × 10^9^/L) vs. non-responders (94 ± 157 × 10^9^/L) (*p*  = 0.033). However, in MDACC research, leukocytosis was not associated with differences in CR rates [5]. The mean ± SD of WBC count in patients who achieved 2-year OS was 16 ± 42 × 10^9^/L whereas in patients who did not achieve 2-year OS was 59 ± 10^9^ × 10^9^/L (*p*  = 0.025). These statistical differences were similar to MDACC results [5]. In addition to WBC count, BMT and myeloid markers also made significant differences in survival outcomes. Despite other studies, the Philadelphia abnormalities have no differences in survival through the current research [3, 5, 7]. Our study limitations were inherent to its small sample size, retrospective model, and lack of long-term follow-up. However, the strength of this study was that it evaluated the Hyper-CVAD regimen in an unselected patient sample.

## 5. Conclusion

Our study found that the results of using the Hyper-CVAD treatment regimen for adult ALL were not as high as we anticipated, which may be due to differences in the groups being studied compared to previous reports. However, the Hyper-CVAD backbone is still considered to be the best option for current and future research and therapy in adult ALL. The flexible backbone of the Hyper-CVAD regimen allows for the easy addition of novel targeted therapies, which may further improve treatment outcomes.

## Figures and Tables

**Table 1:** Baseline characteristics of ALL patients treated with the Hyper-CVAD regimen.

Baseline characteristics (*N* = 70)	
Age mean ± SD (years)	36.61 ± 13.88
Age median [min, max]	36.5 [16, 73]
Gender (%)	
Female	30 (42.9)
Male	40 (57.1)
Cell lineage (%)	
B-cell ALL	54 (77.1)
T-cell ALL	16 (22.9)
Primary CNS involvement (%)	4 (5.71)
Ph+ (%)	8 (18.6)
Myeloid marker (%)	6 (10)
Bone marrow transplantation	16 (22.8)

## Data Availability

The authors confirm that the data supporting the findings of this study are included within the article.
